# Protein Prenylation in Plants: Mechanisms and Functional Implications

**DOI:** 10.3390/plants14121759

**Published:** 2025-06-09

**Authors:** Chang Tian, Quan Wang

**Affiliations:** Shenzhen Branch, Guangdong Laboratory of Lingnan Modern Agriculture, Genome Analysis Laboratory of the Ministry of Agriculture and Rural Affairs, Agricultural Genomics Institute at Shenzhen, Chinese Academy of Agricultural Sciences, Shenzhen 518120, China; tianchang@caas.cn

**Keywords:** protein prenylation, farnesyltransferase, geranylgeranyltransferase, modification mechanism, functional implications

## Abstract

Protein prenylation is a crucial post-translational modification that involves the formation of a covalent bond between isoprenoid lipids and the cysteine residues of specific proteins. This modification plays a significant role in determining protein localization, facilitating protein–protein interactions, and ultimately influencing protein function within the cellular context. Prenylation is a conserved process observed across various kingdoms of life, including plants, animals, fungi, and protists. This review aims to consolidate existing knowledge regarding the mechanisms underlying protein prenylation, encompassing the biosynthetic pathways of isoprenoids in plants and the processing involved in the prenylation modification. Furthermore, it highlights the implications of alterations in protein prenylation on plant development, signaling pathways, and stress responses. The review also addresses the similarities in modification mechanisms between plants and animals, as well as the diversity of their functional implications. Finally, it outlines prospective research directions of the plant prenylation mechanisms and the potential applications in the field of biotechnology.

## 1. Introduction

Protein prenylation is integral to numerous biological processes across diverse organisms. This modification involves the formation of a covalent bond between hydrophobic isoprenoid lipids, such as 15-carbon farnesyl or 20-carbon geranylgeranyl, and specific cysteine residues situated at or near the C-terminus of target proteins. The catalysis of this process is performed by prenyltransferases, which recognize specific amino acid sequence motifs within target proteins. While the isoprenoid modification mechanisms in animals and plants exhibit high conservation in terms of enzymes, substrates, and metabolic pathways, their functional roles are markedly differentiated, reflecting the ecological requirements of various species. In animals, protein prenylation is crucial for regulating development, metabolism, disease processes, as well as reproductive and immune functions [1]. Additionally, this modification is significant in prokaryotic cells; for instance, mutations in *geranylgeranyltransferase* (*IspA*) in *Staphylococcus aureus* result in complete loss of pigmentation and various defects in cellular behavior, including impaired growth and stress responses [2]. This modification appears to be a fundamental process involved in numerous pathways mediating cellular homeostasis, as variations in fatty acid composition and increased membrane fluidity can lead to changes in envelope composition.

In plants, protein prenylation has been recognized as a critical mechanism for facilitating proper growth, development, and adaptation to fluctuating environmental conditions. It is implicated in a broad spectrum of cellular functions, ranging from signal transduction pathways governing plant responses to hormones and environmental stimuli to the maintenance of cellular architecture and organelle organization [3]. Prenylation alone is often insufficient for mediating protein–membrane interactions and protein–protein interactions, which typically require carboxyl-terminal proteolysis, methylation, and either an upstream polybasic domain or a fatty-acylated cysteine residue [3,4,5]. Moreover, there exists a vast array of farnesylated and geranylgeranylated proteins in plants, exhibiting diverse functions, alongside various effects of prenylation and subsequent post-prenylation processes on the targeting and function of different proteins [3,6,7]. These complexities pose challenges in elucidating the physiological significance of protein prenylation and its associated processes.

The investigation of molecular mechanisms, regulatory networks, and functional consequences of protein prenylation in plants is not only of intrinsic scientific significance but also possesses considerable practical relevance. Such research can elucidate plant-specific biological processes, which may be leveraged for agricultural advancements. For example, the manipulation of genes associated with prenylation could facilitate the development of crop varieties with enhanced stress resilience, improved growth traits, and increased yields [8,9]. Furthermore, a deeper comprehension of protein prenylation in plants may enrich our understanding of the evolution of post-translational modification systems across eukaryotes, given that plants exhibit distinct physiological and developmental characteristics compared to animals and fungi. This report seeks to consolidate existing knowledge in this domain, emphasizing recent progress while discussing remaining gaps in our understanding of protein prenylation within plant systems.

## 2. Mechanisms of Protein Prenylation in Plants

The process of protein prenylation in plants is mediated by a group of highly specialized enzymes known as prenyltransferases (PTs). Three major types of PTs have been characterized: protein farnesyltransferase (FTase), protein geranylgeranyltransferase I (GGTase-I), and protein geranylgeranyltransferase II (GGTase-II), all of which are heterodimeric enzymes. FTase and GGTase-I share a common α-subunit, but possess distinct β-subunits that determine substrate specificity. In contrast, GGTase-II is composed of different α- and β-subunits compared to FTase and GGTase-I. These enzymes play a crucial role in catalyzing the addition of prenyl groups to target proteins.

**Figure 1:**
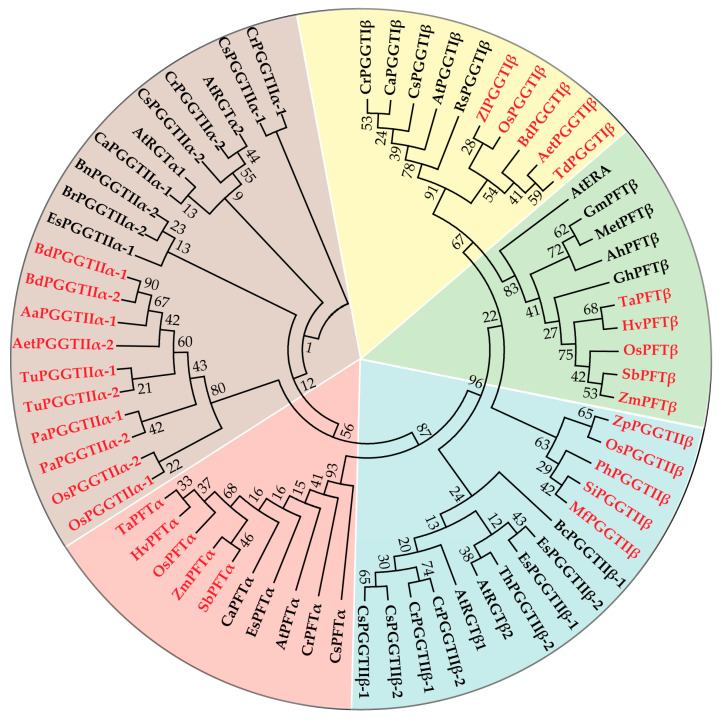
Phylogenetic relationships of prenyltransferase proteins. The full-length amino sequences of prenyltransferase proteins from monocotyledons and dicotyledons plants were used for evolutionary analyses. Monocotyledons: *Oryza sativa* (Os), *Hordeum vulgare* (Hv), *Zizania palustris* (Zp), *Zizania latifolia* (Zl), *Panicum hallii* (Ph), *Setaria italica* (Si), *Miscanthus floridulus* (Mf), *Brachypodium distachyon* (Bd), *Triticum urartu* (Tu), *Triticum dicoccoides* (Td), *Aegilops tauschii subsp. strangulate* (Aet), *Phragmites australis* (Pa), *Alopecurus aequalis* (Aa), *Triticum aestivum* (Ta). *Dicotyledons: Arabidopsis thaliana* (At), *Camelina sativa* (Cs), *Capsella rubella* (Cr), *Eutrema salsugineum* (Es), *Cardamine amara subsp. amara* (Ca), *Raphanus sativus* (Rs), *Brassica carinata* (Bc), *Brassica rapa* (Br), *Brassica napus*, *Tarenaya hassleriana* (Th), *Gossypium hirsutum* (Gh), *Arachis hypogaea* (Ah), *Medicago truncatula* (Met), *Glycine max* (Gm). These analyses were performed in MEGA7 using the Neighbor-Joining method, with the bootstrap value set to 1000 [10]. Evolutionary distances were calculated using the JTT matrix-based method. In the phylogenetic tree, monocotyledonous plants are highlighted in red font, while dicotyledonous plants are indicated in black font. The evolutionary analysis clearly classified these proteins into five distinct groups, each group displayed with a different colored background.

**Figure 2:**
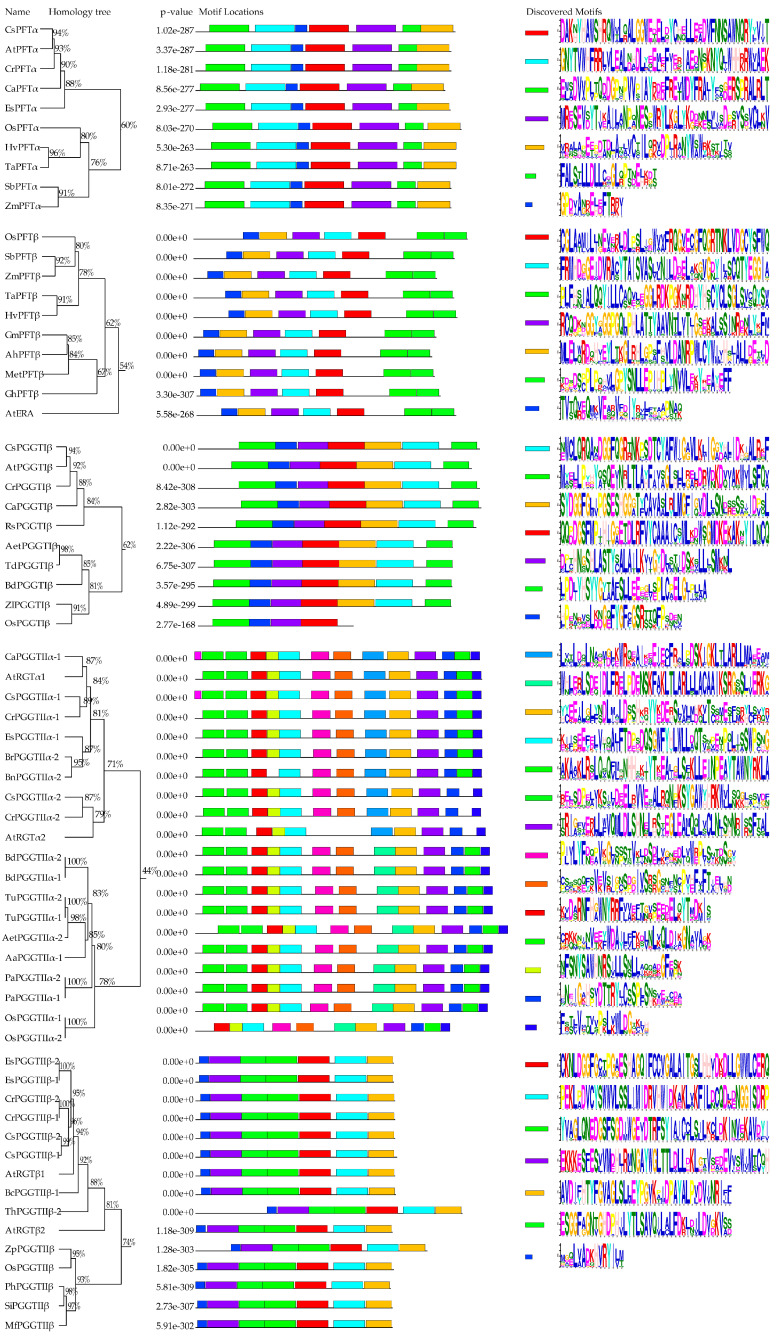
Predicted schematic structures of plant prenyltransferase proteins. The full-length amino acid sequences of prenyltransferase proteins (as shown in Figure 1) were used. In the left section of the figure, the homology analysis of these proteins is displayed. These analyses were performed using the DNAMAN program with the full-length amino acid sequences. The middle section presents the conserved domains identified through the MEME tool [11]. For each protein, the actual motif length and order are indicated. The right section illustrates the motif symbols along with the corresponding consensus sequences for each domain. Sequence LOGOs for each protein domain motif were generated using the MEME algorithm. The height of individual letters within the stack reflects their probability of occurrence at that specific position, multiplied by the total information content of the stack.

A total of 65 prenyltransferase proteins were selected for systematic evolutionary relationship analysis, including 30 from monocotyledonous plants and 35 from dicotyledonous plants (Figure 1). The evolutionary analysis clearly classified these proteins into five distinct groups based on the phylogenetic positions of different prenyltransferase subunits, with evident evolutionary boundaries between monocotyledonous and dicotyledonous plants. The prenyltransferase proteins shown in Figure 1 were subsequently analyzed for homology comparison and protein motif identification (Figure 2). Full-length sequence analysis revealed that prenyltransferase proteins, including FTase β subunits, showed homology levels exceeding 50%. FTase α and GGTase-I α subunits demonstrated over 70% homology, while GGTase-I and GGTase-II β subunits exhibited even higher homology, surpassing 80%. In Arabidopsis, two GGTase-II α subunits (RGTA1: At4g24490; RGTA2: At5g41820) and two β subunits (RGTB1: At5g12210; RGTB2: At3g12070) were identified. BLASTP searches using these full-length amino acid sequences against the NCBI database revealed highly similar homologous genes in *Oryza sativa* (Os), *Brachypodium distachyon* (Bd), *Triticum urartu* (Tu), *Phragmites australis* (Pa), *Eutrema salsugineum* (Es), and *Capsella rubella* (Cr). Only one homologous gene for RGTA and RGTB was found in these species, as evidenced by shared gene accession numbers (e.g., *BdPGGTIIα-1/BdPGGTIIα-2*, *TuPGGTIIα-1/TuPGGTIIα-2*, *PaPGGTIIα-1/PaPGGTIIα-2*, *OsPGGTIIα-1/OsPGGTIIα-2*, *EsPGGTIIβ-1/EsPGGTIIβ-2*, *CrPGGTIIβ-1/CrPGGTIIβ-2*). Furthermore, the motif structure of each prenyltransferase subunit was found to be conserved across both monocotyledonous and dicotyledonous plants. The phylogenetic analysis, consistent with homology comparisons and domain architecture, further underscores the evolutionary conservation of protein function while revealing species-specific characteristics.

**Figure 3:**
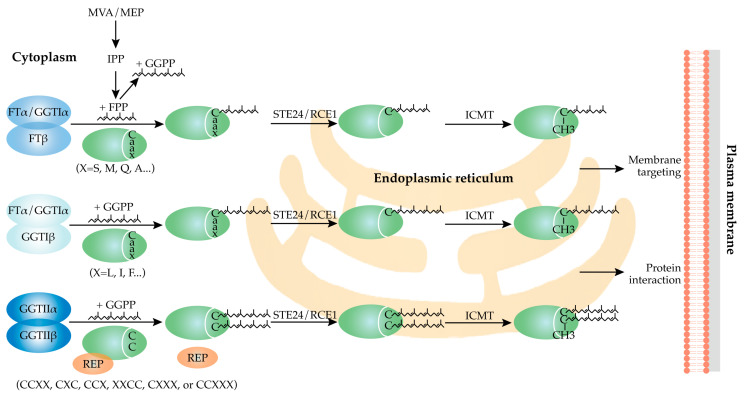
Protein Prenylation in Plants. The figure depicts the structure of CAAX protein prenyltransferases (including FTase and GTase), their substrate preference for a C-terminal motif, the processing steps of prenylated protein modification, and the final form of the prenylated substrate.

FTase is responsible for transferring a 15-carbon farnesyl moiety from farnesyl pyrophosphate (FPP) to substrate proteins, while GGTase-I facilitates the bond formation of a 20-carbon geranylgeranyl moiety derived from geranylgeranyl pyrophosphate (GGPP) (Figure 3) [4,7,12,13]. FPP and GGPP are the trimer and tetramer formed after the condensation reaction of isopentenyldiphosphate (IPP) and dimethylallyldiphosphate (DMAPP) [14,15]. IPP and DMAPP are produced via two distinct metabolic pathways, namely the mevalonate (MVA) pathway and the methyl-erythritol 4-phosphate (MEP) pathway [14]. The MVA pathway, located in the plant cytoplasm, is primarily involved in the biosynthesis of brassinosteroids, sesquiterpenes, sterols, and triterpenes, whereas the MEP pathway, occurring in plastids, is chiefly responsible for the synthesis of abscisic acid (ABA), gibberellins, chlorophyll, carotenoids, monoterpenes, and diterpenes [16,17]. The flux of the FPP and GGPP biosynthetic pathways can influence the efficiency of prenylation. HMG-CoA reductase (HMGR) catalyzes the rate-limiting step of the MVA pathway, and its activity directly determines the supply of FPP/GGPP. HMGR is regulated by the cell’s energy status (AMP-activated SNF1-like kinase can inhibit HMGR), thereby affecting the local generation amount of FPP/GGPP. When sucrose supply is sufficient, HMGR activity increases, promoting GGPP synthesis, causing CaM53 protein to geranylgeranylate and localize to the plasma membrane; conversely, when there is no sucrose, HMGR is inhibited, and the unmodified CaM53 accumulates in the nucleus due to nuclear localization signals [18].

**Figure 4:**
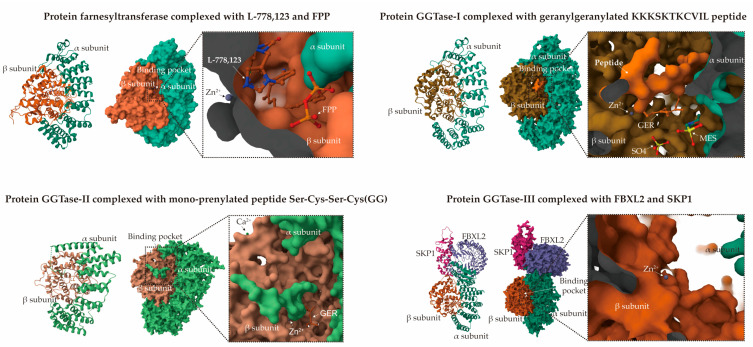
Structural overview of prenyltransferase heterodimers. Farnesyltransferase (FTase) (PDB ID: 1S63), geranylgeranyltransferase I (GGTase-I) (PDB ID: 1N4R), GGTase-II (PDB ID: 3DSV), and GGTase-III (PDB ID: 6O60). The α subunits are rendered in green color gradients, while β subunits are presented in orange gradients. Each subpanel is divided into three sections: the helical structure in cartoon representation, molecular surface, and magnified views of the binding pockets. All heterodimers display Zn^2+^ coordination, with additional Ca^2+^ coordination observed specifically in the GGTase-II polymer. Key molecular components—including Zn^2+^ ions, farnesyldiphosphate (FPP), and the prenylated peptide—are explicitly indicated by arrows within their respective binding pockets.

The prenylation mechanisms of FTase and GGTase-I are characterized by the recognition of specific amino acid motifs within substrate proteins, typically featuring a CAAX motif at the C-terminus (Figure 3) [3,4,7,19]. In this motif, ‘C’ denotes cysteine, while ‘A’ signifies an aliphatic amino acid. The identity of ‘X’ is critical in determining whether the protein undergoes farnesylation or geranylgeranylation. Specifically, when ‘X’ is serine, methionine, glutamine, or alanine, farnesylation is favored; conversely, if ‘X’ is leucine, isoleucine, or phenylalanine, geranylgeranylation is more likely to occur. Prenylation is catalyzed by PFTs, which facilitate the transfer of the prenyl moiety to the cysteine residue within the CAAX motif, resulting in the formation of a thioether bond that covalently links the hydrophobic lipid to the protein. The zinc finger structure found in the β subunit of FTase and GGTase-I, which requires Zn^2+^ for maintaining inter-subunit interactions and the conformational stability of the substrate-binding domain, plays an indirect role in substrate recognition (Figure 4). Additionally, the catalytic activity of both transferases is dependent on the presence of Mg^2+^. FPP/GGPP binds to the binding site through hydrophobic and electrostatic interactions. The hydrophobic interactions primarily originate from aromatic residues surrounding the farnesylation substrate, while the polar interactions result from multiple hydrogen bonds formed between the farnesylation substrate and the protein [20,21]. The catalytic mechanism of the prenylation reaction begins with the preferential binding of FPP or GGPP to the specific pocket of FTase/GGTase-I enzymes through electrostatic interactions (coordinated with Mg^2+^) and hydrophobic interactions (with aromatic residues in the enzyme’s active center) [22]. Under the action of prenyltransferase, the thiol group (-SH) of the cysteine residue in the C-terminal CAAX motif of the protein is deprotonated to form a thiolate anion (S^−^), which acts as a nucleophile to attack the electrophilic carbon (C-1) of farnesyl or geranylgeranyl pyrophosphate, resulting in the formation of a thioether bond (C-S-C prenyl) while releasing pyrophosphate (PPi). The active site of the enzyme (such as the Zn^2+^ site in FTase) polarizes the thiol group of Cys to enhance its nucleophilicity. The dissociation of the prenylated product requires the binding of a new substrate molecule (FPP/GGPP), which, through allosteric effects, pushes the modified protein into the exit groove (Figure 4). Protein prenylation can induce conformational rearrangement through hydrophobic interactions of the prenyl group [21,22]. This enables the prenyl group to insert into the cell membrane’s lipid bilayer and facilitates membrane anchoring. Additionally, the prenylated cysteine residues are often carboxymethylated, a modification that neutralizes their negative charge and enhances membrane affinity [5].

In contrast to FTase and GGTase-I, GGTase-II requires an auxiliary subunit, Rab Escort Protein (REP), to present Rab GTPase substrates to the catalytic core [23,24]. Recent studies have also indicated that Arabidopsis can prenylate certain non-Rab GTPases independently of REP (Figure 3) [25]. The existing literature suggests that GGTase-II requires a more complex C-terminal protein sequence, which may include one or two cysteine residues in the target protein, exemplified by motifs such as CCXX, CXC, CCX, XXCC, CXXX, or CCXXX [3,4,26]. RABA1A, RABA2A, RABF2A, and RABG2, which contain the C-terminal consensus sequences CCSN, CCSSS, SCCA, and GCAC, respectively, were prenylated by GGTase-II (RGTA1·RGTB1) in the presence of AtREP in vitro. All double-cysteine-substitution mutant proteins remained unprenylated, while single-cysteine-substitution mutant proteins exhibited some degree of prenylation, indicating that in all four types of C-terminal sequences, either cysteine residue is capable of undergoing geranylgeranylation [25]. Unlike FTase, GGTase-I does not require Mg^2+^ for its enzymatic activity [27].

After prenylation, the modified substrate protein undergoes further processing. The AAX residues are often proteolytically removed by one of two CaaX endoproteases, FACE1/STE24 and FACE2/RCE1, after which the newly exposed isoprenylcysteine residue is methylated by one of two isoprenylcysteine methyltransferases (ICMTs) (Figure 3) [23,28]. These additional protein hydrolysis and methylation steps further modify the hydrophobicity and function of the prenylated protein. In yeast and animal cells, the CAAX endoproteases and ICMTs are localized in the ER membranes, while prenyltransferases are found in the cytoplasm [4,29,30]. This localization pattern is conserved in plants as well [3,28,31,32], suggesting that prenylated proteins are initially modified in the cytoplasm and subsequently processed within the ER system. Furthermore, carboxyl methylation is a reversible and potentially regulated step in the post-translational modification of prenylated proteins. Isopentenylcysteine methylesterase (ICME) catalyzes the demethylation of isopentenylcysteine methylester [33]. The prenylation process is also related to other post-translational modifications, such as ubiquitination and palmitoylation, although research in this area remains limited. For instance, in Arabidopsis, AGG2 undergoes both prenylation and palmitoylation [34]; MUBs are ubiquitination-related proteins, membrane-anchored by farnesylation, geranylgeranylation, or palmitoylation [35]. The simultaneous occurrence of prenylation with other post-translational modifications contributes to stable membrane binding and improves substrate modification functions.

Protein prenylation in plants specifically mediates the formation of covalent bonds between farnesyl or geranylgeranyl isoprenoid groups and the cysteine residue located in the C-terminal CAAX motif of substrate proteins. This process, catalyzed by Mg^2+^ and assisted by Zn^2+^, culminates in sequential proteolytic cleavage and methylation, thereby facilitating protein maturation and the subsequent regulation of membrane localization and function (Figure 3 and Figure 4). This post-translational modification is critically conserved across eukaryotes and plays a pivotal role in governing cellular signaling cascades, membrane transport, and stress adaptation. Although the core enzymatic machinery exhibits homology with animal systems, plant-specific divergences in substrate selectivity and regulatory networks likely reflect adaptive evolution in response to distinct physiological demands.

## 3. Functional Implications of Protein Prenylation in Plants

Prenylation plays a crucial role in regulating various cellular processes, which integrate cellular homeostasis, developmental control, and environmental responses through protein interactions and enzymatic activity. One of the primary functions of protein prenylation in plants is to modify the subcellular localization of proteins, particularly their association with cellular membranes. The hydrophobic characteristics of the prenyl moiety facilitate the anchoring of prenylated proteins to membranes, such as the plasma membrane and the endoplasmic reticulum (ER) [4]. For example, DnaJ, a protein found in a complex with the chaperone Heat Shock Protein 70 (Hsp70), requires farnesylation for membrane binding [36]. Prenylated CaM53 localizes to the plasma membrane, whereas the unmodified CaM53 accumulates in the nucleus [5]. In wild-type plants, AGG1 and AGG2 are associated with plasma membranes, but their membrane localization is disrupted in mutants lacking both PFT and PGGT-I activity [34]. This membrane association is crucial for the proper functioning of many prenylated proteins, as it enables them to participate in signaling complexes and interact with other membrane-bound proteins.

Prenylated proteins are particularly important for plant adaptation to both abiotic and biotic stresses (Table 1 and Table 2). In abiotic stress responses, they are essential for enhancing drought tolerance (e.g., farnesylated HSP40 related ABA signaling for stomatal closure and drought tolerance), salt resistance (e.g., overexpression of *OsRab7*, a geranylgeranylated GTPase, enhances salt tolerance), heavy metal detoxification (e.g., farnesylated HIPP proteins such as CdI19 binding and sequestering cadmium), and high temperature tolerance (e.g., prenylated DnaJ1 stabilizing membrane proteins under high temperatures). In biotic stress responses, they mediate defense against pathogens, such as fungal resistance (e.g., the *AGG1*-deficient mutant exhibits decreased resistance to necrotrophic pathogens, reduced expression of the plant defensin PDF1.2, and hypo-sensitivity to methyl jasmonate; HIPP1 and ROP6 are related to powdery mildew fungus defense).

### 3.1. Protein Prenyltransferase

The expression of genes encoding FTase, GGTase-I, and GGTase-II enzymes can be modulated by developmental signals, environmental factors, and hormonal influences, leading to variations in their abundance and enzymatic activity. The functions and phenotypes of genes related to prenylation modification in Arabidopsis are summarized in Table 1. In Arabidopsis, the *ERA1* gene, which encodes the β-subunit of FTase, is predominantly expressed in meristematic regions, guard cells, and floral tissues, reflecting its involvement in meristem development and stomatal closure [37,38]. The *era1* mutant exhibits enlarged meristems and extra floral organs. Loss-of-function mutations in the Arabidopsis *PLP* gene (FT/GGT I-α) result in significantly larger meristems and an increased number of floral organs, particularly petals [39]. Mutants of the *GGT I-β* subunit (*ggb*) do not display pronounced developmental phenotypic variations, but double mutants of *era1*/*ggb* exhibit phenotypic defects similar to those in *plp* mutants [40]. Notably, overexpression of *GGB* can ameliorate the phenotypic manifestations associated with the *era1* mutation [40]. Additionally, *ERA1* affected branching development in Arabidopsis under short-day conditions, particularly by reducing the number of branches at the cauline-axil, as these axillary structures failed to differentiate rather than being inhibited in meristematic tissue development [37]. Together, these observations establish the role of prenyltransferases in regulating meristem initiation and development, as well as the functional redundancy between *FTase* and *GGTase-I*. In contrast to Arabidopsis, homozygous mutants of *OsFT*/*OsGGT I*-*α* in rice exhibit a lethal phenotype, while heterozygous mutants show reduced pollen viability [31]. *OsFT*/*OsGGT I*-*α*, *OsFT*-*β*, and *OsGGT I*-*β* are expressed broadly across various tissues, including panicles, leaf blades, sheaths, roots, and floral organs (anthers, stigmas, and ovaries) [31]. These genes exhibit higher expression levels in leaf blades. Notably, *OsFT*/*OsGGT I*-*α* displayed significantly higher transcript abundance in floral organs compared to *OsFT*-*β* and *OsGGT I*-*β*, suggesting the potential roles of different subunit levels in the output of protein prenylation.

**Table 1:** The function and phenotype of prenylation modification-related genes in Arabidopsis.

Gene	Function/Phenotype	Hormone-Related Regulation
*ERA1* (*FTase*-*β*)	1 Predominantly expressed in meristematic regions, guard cells, and floral tissues; 2 Mutant (*era1*): enlarged meristem, increased flower organs, and reduced branches under short days; 3 Regulate the development of meristematic tissues and the closure of stomata; 4 With *GGB* functional redundancy (double mutant *era1*/*ggb* phenotype similar to *plp* mutant);	1 Negative regulation of ABA signaling (*era1* mutant is sensitive to ABA); 2 Promote lateral root formation by reducing *ABI3* expression; 3 No significant impact on auxin-inhibited primary root growth.
*PLP* (*FT*/*GGT I*-*α*)	Mutant (*plp*): significantly larger meristems and an increased number of floral organs, particularly petals	The double mutant *era1*/*plp* was less sensitive to external ABA than *era1* plants themselves
*GGB* (*GGT I*-*β*)	1 The single mutation shows an insignificant phenotypic difference, but exhibits a similar *PLP* defect to the *era1* double mutation; 2 Overexpression of *GGB* can partially rescue the *era1* phenotype.	1 Enhance auxin-induced lateral root formation; 2 Negative regulation of ABA signal (sensitive only in stomatal response, does not affect seed germination).
*ICMT*/*ICME*	1 *ICMTox* and *ICMEox* plants, similar to *ggb* mutants, showed no developmental defects; 2 *ICMT*: inhibits ABA signal (overexpression leads to ABA insensitivity); 3 *ICME*: promotes ABA signal (overexpression leads to ABA hypersensitivity); 4 *ICME* is induced by ABA, forming a positive feedback regulation.	Related to ABA signal directly, but has no significant correlation with auxin signal (NAA does not change *ICME* expression).

Protein prenyltransferases also play critical roles in hormone signaling pathways, such as ABA [41] and auxin. The phenotypic characteristics of the *era1* mutant, including apical meristem enlargement, increased lateral root formation, and defects in lateral branch initiation, may also be associated with alterations in auxin signaling [37,42]. *ABI3* is a transcriptional activator that positively regulates both ABA and auxin signaling pathways. The enhanced lateral root formation in *era1* mutants is likely mediated by reduced *ABI3* expression [42]. Similarly, *ggb* mutants also exhibit increased lateral root formation in response to auxin treatment [40]. However, these mutants show no significant impact on auxin-inhibited primary root growth. Knockout mutations in *ERA1* confer sensitivity to ABA in both seeds and stomata [40,42]. Likewise, *ggb* mutants demonstrate heightened ABA responsiveness in stomatal regulation but, unlike *era1* mutants, do not exhibit significant changes in ABA sensitivity during seed germination [40,42]. Therefore, it can be concluded that prenylated proteins serve as negative regulators of ABA signaling.

Membrane association and function of prenylated proteins in Arabidopsis are influenced by prenylcysteine methylation and demethylation processes. Isoprenylcysteine methyltransferase (ICMT) serves as a negative regulator of ABA signaling, whereas isoprenylcysteine methylesterase (ICME) acts as a positive regulator [43]. Overexpressed *ICMT* lines exhibit ABA insensitivity in stomatal closure and seed germination assays, while overexpressed *ICME* lines show ABA hypersensitivity. The expression of the *ICME* gene is induced by ABA, leading to demethylation and subsequent inactivation of prenylated negative regulators in the ABA signaling pathway, thereby establishing a positive feedback loop for ABA signaling [43]. Notably, application of naphthaleneacetic acid (NAA) does not significantly alter *ICME* mRNA abundance. *ICMTox* and *ICMEox* plants, similar to *ggb* mutants, showed no developmental defects, which contrasts with the enlarged meristems and floral organ proliferation observed in *era1-2* and *plp* mutants [40,42,43]. This observation may indicate that meristematic isoprenylated proteins remain fully methylated despite *ICME* overexpression, potentially due to high ICMT activity, or that their functionality is independent of methylation/demethylation processes. Importantly, *clv3-7* mutants, which exhibit similar meristematic defects, show reduced expression of *STE14A* (*ICMT*), suggesting a role for *ICMT* in regulating meristem development [43]. These findings highlight the role of protein prenylation in mediating interactions between ABA and auxin signaling pathways.

### 3.2. Prenylated Proteins

Existing data show the existence of numerous isoprenylation-modified proteins in plants. For example, a study using [^3^H]-radiolabeled precursors of the mevalonate pathway to tag isoprenylated proteins in spinach seedlings revealed that all cellular fractions contain such proteins [44]. The majority are localized in mitochondria and nuclei, though their levels in the cytosol and chloroplasts are relatively low. Protein prenylation intersects with various biological processes, including the cell cycle, microRNA regulation, metabolic regulation, stress responses, and hormone signaling pathways, as summarized in Table 2. This intersection influences both cellular and whole-plant processes, with significant implications for plant growth and development. However, the specific functional roles of protein prenylation in plants remain inadequately understood. Elucidating its substrate specificity and characterizing regulatory networks involving interacting partners are expected to provide critical insights into this post-translational modification mechanism in plant systems.

**Table 2:** Functional characterization of prenylated proteins in plants.

Stress/ Growth	Gene	Plant ^a^	Partial Gene ID (Genebank, NCBI, Tair)	Definition	Function	Modification ^b^
Plant growth and development	*APETALA1*	Arabidopsis	Z16421	MADS box transcription factor	Floral meristem identity, sepal and petal identity development	F [38]
	*AtIPT3*	Arabidopsis	AT3G63110	Pentenyltransferase, cytokinin synthase	Cytokinin biosynthesis	F [45]
	*AtNAP1;1*	Arabidopsis	At4g26110	Nucleosome assembly protein 1	Cell proliferation and expansion	F [46]
	*AUX2–11 (IAA4)*	Arabidopsis	L15450	AUX/IAA family of transcriptional repressors	Auxin signaling	GG [47]
	*ROP1*	Arabidopsis	AT3G51300	Rho GTPases	Tip growth of pollen tubes	GG [48]
Plant growth and development; abiotic stress; biotic stress	*RABs*	Arabidopsis	U46925 etc.	Rab GTPases	Vesicular transport, and ethylene signaling, plant development and environmental stress adaptation	GG [25,49,50,51,52,53]
abiotic stress	*J2; J3*	Arabidopsis	AT5G22060; AT3G44110	HSP40 proteins DnaJ homologues	Heat stress; small RNA-mediated gene regulation	F [54,55]
	*DnaJ1*	*Atriplex nummularia*	P43644; AJ299254	DnaJ-like heat shock chaperones	Response to high temperature, NaCl stress, and ABA signaling	F [36]
	*DnaJ1*	Tobacco; tomato etc.	AJ299254; XP_004231500 etc.	DnaJ-like heat shock chaperones	Drought response and ABA signaling	P [56,57,58]
	*CYP85A2*	Arabidopsis	AT3G30180	Cytochrome P450 enzyme	Brassinolide accumulation, ABA response, and drought tolerance	F [59]
	*PhCaM53*	*Petunia x hybrida*	M80831	Calmodulin-like proteins	Calcium signal transduction	F, GG [60]
	*OsCaM61*	*Oryza sativa*	U37936			P [61]
	*ATFP3*	Arabidopsis	U64906	CCH copper chaperone-related	Response to heavy metals	F [62]
	*Cdl19*	Arabidopsis	AF517549	Cd-induced genes	Response to heavy metals	P [63]
	*AtRAC7*	Arabidopsis	AF079484	Rho GTPases	Signal transduction, cytoskeleton morphogenesis, ROS production, and hormone response	F, GG [64]
biotic stress	*HIPP1*	Wheat	AKB91757.1	Heavy metal-associated isoprenylated plant protein	Powdery mildew resistance	P [8]
	*AGG1*; *AGG2*	Arabidopsis	At3g63420; At3g22942	Heterotrimeric G proteins Gγ subunit	ABA, auxin signaling, plant defense	F, GG [34]
	*ROP6*	Arabidopsis	OAP00270	Rho GTPases	Developmental and pathogen response signaling	F. GG [65,66,67]
	*MUB1,3-6*	Arabidopsis	At3g01050 etc.	Ubiquitin-fold proteins	Unknown	F, GG [35]
Unknown	*GmFP1; 2; 3*	*Glycine max*	U13179-U13181	*Glycine max* farnesylated protein	Unknown	F [68]

^a^ Partial prenylation-modified proteins, represented by *Arabidopsis thaliana* as a model study. ^b^ Farnesylated and/or geranylgeranylated. Farnesylation is denoted by “F”, geranylgeranylation by “GG”. Prenylation is denoted by “P”, indicating conclusions inferred from experimental results that have not been verified through direct protein modification experiments.

Phylogenetic analyses of small GTP-binding proteins in Arabidopsis indicate the presence of distinct families of GTPases, including Rab, Rho, Arf, and Ran, implicated in various cellular processes such as signal transduction, cell proliferation, cytoskeletal organization, and intracellular membrane trafficking [69]. Several members of the RAB and RHO families have been demonstrated to undergo prenylation modifications in vivo and/or in vitro, including RABA1A, RABA2A, RABF2A, RABG2, Rop1Ps, ROP6, and RAC7 [25,65]. In Arabidopsis, 54 out of 57 Rab GTPases possess C-terminal cysteine residues, making them potential candidates for prenylation, with the exception of RabA4e, RabE1b, and RabF1, which lack a cysteine terminus [25]. RABs are also involved in various functions, including cell wall composition [70], polarized growth of root hairs and pollen tubes [71], endocytic sterol trafficking [72], and vacuolar trafficking [53]. Small GTPases like ROPs (Rho of plants) depend on geranylgeranylation for their localization to the plasma membrane, thereby influencing polar growth and stress responses [73]. The *Atrop6^DN^* mutant exhibits a range of developmental abnormalities, including small and multiple inflorescence stems, twisted leaves, deformed pavement cells in the leaf epidermis, and altered cytoskeletal organization [65]. Furthermore, *rop6^DN^* plants demonstrate enhanced preinvasive defense responses against a host-adapted virulent powdery mildew fungus but compromised defenses against a non-adapted powdery mildew. The developmental defects and sensitivity to powdery mildew associated with ROP6 appear independent of the salicylic acid (SA)-associated response. Rop1Ps, a geranylgeranylated Rop GTPase, is highly concentrated in the cortical region of the tube apex and the periphery of the generative cell [48]. It may play a role in the signaling mechanism regulating actin-dependent tip growth in pollen tubes.

Recent studies have demonstrated that protein farnesylation significantly influences the functional regulation of microRNAs (miRNAs). In Arabidopsis, the expression of *miRNAs* associated with abiotic stress, which are regulated by the transcription factor SPL7, relies on the farnesylation of HSP40 (J2/J3). Notably, farnesyltransferase mutants and the *J3^C417S^* farnesylation-deficient line showed a marked decrease in the levels of *pri-miR397a*, *pri-miR398b*/*c*, and *pri-miR857* [74]. Furthermore, farnesylation enhances the membrane association of J2/J3 and their interaction with ARGONAUTE 1 (AGO1) while suppressing miRNA binding to membrane-bound polysomes (MBPs) [55]. miRNAs, such as *miR166*, were significantly enriched in MBP fractions in *era1–2* mutants [35]. Disruptions in the HSP40/HSP70/HSP90 system may differentially affect the loading of RNA-induced silencing complexes (RISCs) [75]. It is important to note that miRNA-mediated mRNA cleavage, at least for certain miRNAs, and translational repression predominantly occur on the rough endoplasmic reticulum [76], with cleavage and translation processes being coupled [77,78]. In plants, numerous miRNAs, including *miR164* and *miR166*, have been identified as regulators of growth and development. Overexpression lines of these miRNAs and loss-of-function mutants of their target genes display phenotypes partially similar to those observed in *era1-2* and *plp* mutants, such as defects in meristem development [55,59,79,80,81,82,83]. In comparison to single mutants *dcl1–11* and *era1–2*, the *dcl1–11*/*era1–2* double mutants exhibited developmental abnormalities including cup-shaped cotyledons, filamentous floral structures replacing normal flowers, and trumpet-shaped leaves [55]. These phenotypes resemble those seen in mutants affecting the *miR165*/*miR166*-binding site of *REVOLUTA* (*REV*) [84]. While certain direct targets of *REV* were upregulated in *dcl1–11*/*era1–2* double mutants compared to individual single mutants, this pattern was not universal for all transcription factor targets repressed by miRNAs. Double mutants of *ago1–27*/*era1–2* exhibited severe growth reduction compared to *ago1–27* alone and were completely sterile, which was distinct from either single mutant [55]. Although these genetic interactions do not provide conclusive molecular evidence linking protein farnesylation with miRNA-mediated regulation, they strongly suggest that protein farnesylation participates in developmental processes associated with and potentially modulated by the miRNA pathway.

Various developmental phenotypes have been identified in mutants of prenyltransferases. These phenotypic alterations, including changes in leaf arrangement and an increase in the number of floral organs, can be attributed to the expanded and disorganized meristems observed in *era1* and *plp* mutants [85]. The transcription factor APETALA1 (AP1), which possesses a CFAA motif, plays a crucial role in regulating the transition from inflorescence shoots to floral meristems, as well as the development of sepals and petals. The ectopic expression of a farnesyl cysteine–acceptor mutant of AP1, designated ap1mS, failed to promote the development of compound terminal flowers and instead led to the emergence of novel phenotypes in Arabidopsis. Similarly, compound terminal flowers did not develop in *era1-2* transformants that ectopically expressed *AP1* [38]. This observation suggests that farnesylation modification is essential for the proper function of AP1 in floral meristems. In addition, AP1 regulates cytokinin levels by directly suppressing the cytokinin biosynthetic gene *LONELY GUY1* (*LOG1*) and activating the cytokinin degradation gene *CYTOKININ OXIDASE*/*DEHYDROGENASE3* (*CKS3*) [86]. A truncated version of AP1 that removed the C-domain was unable to rescue the phenotypes of the *ap1* and *ap1-1 cal1-1* double mutant and lost most of the specific interactions associated with AP1, along with some interactions common to both AP1 and CAL [87]. The absence of farnesylation modification may be one of the reasons affecting the interaction and biological function of AP1. Whether the direct regulation of cytokinin levels by AP1 depends on farnesylation modification still requires further investigation.

Mutants of *era1* and *ggb* show increased responsiveness to ABA in stomatal regulation [40,42]. Genetic epistasis studies involving *abi2* mutants confirm that FTase acts upstream of ABA-responsive calcium channels [88]. *ea1* mutants exhibit enhanced drought resistance, which is attributed to hypersensitive stomatal closure mediated by ABA-induced calcium signaling [89]. ABA causes AtRac1 (Rho GTPases) to transition from an active GTP-bound state to an inactive GDP-bound state, and the inactive AtRac1 triggers the disassembly of the actin cytoskeleton in guard cells [90]. This process is dependent on the upstream signaling component ABI1. AtRAC1 contains a C-terminal protein sequence predicted to undergo prenylation, CSIL (GenBank accession: U62746). In cellular contexts, active GTP-bound Rho GTPases localize to the plasma membrane via their carboxy-terminal prenyl groups. Upon inactivation, these GTPases are extracted from the membrane by RhoGDI (Rho guanine nucleotide dissociation inhibitor), which sequesters the prenyl moiety and keeps the inactive GTPase in a soluble cytosolic state [91]. Therefore, plants may regulate stomatal closure to maintain water balance through the ABA-mediated prenylation modification pathway of AtRAC1, but how ABA regulates prenylation modification remains to be studied.

In addition to ABA signals, prenylation modification is necessary for the normal regulation of auxin signaling, cytokinin biosynthesis, pathogen resistance, and other fundamental processes. In Arabidopsis, the initial step in cytokinin biosynthesis is catalyzed by adenosine phosphate-isopentenyltransferases (AtIPTs) [92]. These enzymes are localized in plastids or the cytoplasm and utilize dimethylallyl-diphosphate derived from the methylerythritol phosphate or mevalonic acid pathways. Specifically, AtIPT3 enhances the synthesis of isopentenyl-type cytokinins and serves as a substrate for protein farnesyl transferase, with farnesylation influencing the protein’s localization to either the nucleus/cytoplasm or plastids, depending on its farnesylated or non-farnesylated state [45]. Gain-of-function mutants revealed that the distinct subcellular localization of farnesylated/nonfarnesylated AtIPT3 correlates with the preferential synthesis of isopentenyl- or zeatin-type cytokinins. Mutation of farnesylation site cysteine-333 abolished cytokinin production, indicating the dual necessity of farnesylation and catalytic activity [45]. Rab GTPases, which are modified by Rab-GGTase (GGTase-II), use double cysteine motifs (e.g., XXCC) for membrane association, thereby facilitating vesicle trafficking in stress responses [51,93]. *McRAB5b* expression, a Ypt/Rab GTPase from *Mesembryanthemum crystallinum*, is induced by salt stress [51]. Heterologous expression in *E. coli* generated antibodies, revealing that membrane-associated McRAB5b exists as monomers and dimers in vitro/vivo. Only the monomeric form is capable of binding GTP, suggesting that its activity may be regulated through transitions between monomeric and dimeric states [51]. Additionally, AtFP3 and Cdl19, which are farnesylated at their CAAX motifs, facilitate heavy metal detoxification [62,63]. FPs is a novel family of plant prenylated proteins with metal-binding motifs (M/LXCXXC core) identified in Arabidopsis, soybean, and tobacco [62]. Exemplified by Arabidopsis AtFP3, which carries a functional CaaX prenylation site, it demonstrated efficient in vitro prenylation and binding of transition metal ions (Cu^2+^, Ni^2+^, Zn^2+^) [62]. In vivo labeling with [^14^C]-mevalonate in tobacco BY2 cells confirmed the presence of soluble, prenylated proteins that bind to copper-IMAC columns, indicating their widespread occurrence in plants [62]. CdI19, a metal-binding protein in Arabidopsis, contains a consensus prenylation site at its carboxy-terminus. This protein binds Cd through its CXXC motif, localizes to plasma membranes (as shown by GFP fusion in BY2 cells), and its transcription is induced by Cd^2+^, Hg^2+^, Fe^2+^, and Cu^2+^ [63]. Overexpressing *CdI19* enhanced Cd tolerance, suggesting its role in heavy metal homeostasis/detoxification by acting as a plasma membrane barrier against intracellular heavy metal influx [63]. RABs are also involved in ethylene signaling transduction [52]. Ethylene rapidly and transiently up-regulates the activity of several *Rabs*, such as *Rab8* and *Ara3* [52]. This activation is suppressed by the receptor-directed inhibitor 1-methylcyclopropene. Moreover, impaired farnesylation of cytochrome P450 CYP85A2 reduces brassinosteroid biosynthesis (BR), although the farnesylation-deficient *cyp85a2* mutant exhibits lower ABA sensitivity compared to *era1* mutants [59]. The farnesylation site of CYP85A2 is not conserved, suggesting the presence of additional farnesylation targets that critically regulate ABA signaling and drought tolerance beyond CYP85A2 [59]. Membrane-anchored ubiquitin-fold proteins (MUBs), characterized by C-terminal CaaX sequences, are conserved across plants (e.g., Arabidopsis, rice), animals, and fungi [35]. In Arabidopsis, MUBs undergo lipid modifications, including farnesylation (AtMUB1/4), geranylgeranylation (AtMUB3/5/6), or palmitoylation (AtMUB2) for membrane attachment. Although the specific functions of MUBs remain largely undefined [3], ubiquitin-fold proteins are known to play significant roles in various cellular processes such as protein degradation [94], SCF complex activation [95], and autophagy [96]. HIPP1-V positively regulates wheat resistance against powdery mildew (caused by *Blumeria graminis f.sp. tritici*, *Bgt*) [8]. Infection by *Bgt* rapidly induces the expression of *HIPP1-V*, and its transient/stable overexpression in wheat suppresses pathogen haustorium formation and enhances resistance. The prenylation of HIPP1-V is crucial for its localization to the plasma membrane, its interaction with the E3 ligase CMPG1-V, and its overall function [8]. The non-prenylated HIPP1-VC^148G^ mutant failed to confer resistance. Transcriptomic analyses have established a connection between reactive oxygen species [95] and SA pathways in relation to HIPP1-V-mediated resistance [8].

## 4. Commonality of Plant and Animal Modification Mechanisms and the Diversity of Their Functions

This prenylation modification is conserved in plants, animals, fungi, and protists. *AtSTE24*, *AtRCE1*, *AtICMTA*, and *AtICMTB* from Arabidopsis could functionally complement related yeast mutants, indicating the evolutionary conservation of all parts of this processing machinery [28]. In animals, similar to plants, there exist FTase, GGTase-I, and GGTase-II, which exhibit comparable substrate recognition specificity and modification mechanisms [97]. In Arabidopsis, the β subunits (ERA1: At5g40280, GGB: At2g39550) and shared α subunit of FTase and GGTase-I (PLP: At3g59380) are present in single copies [3]. Compared to yeast and animals, GGTase-II is less well characterized in plants. In Arabidopsis, two putative GGTase-II α and β subunits (RGTA1: At4g24490, RGTA2: At5g41820, RGTB1: At5g12210, RGTB2: At3g12070) have been identified, along with an REP (At3g06540) homolog [3,23,24]. Current examinations indicate that mammals possess single copies of the α and β subunits [26,98]. Additionally, two isoforms of REP, REP-1 and REP-2, are known to be ubiquitously expressed in mammals [99]. In yeast, only a single essential gene has been identified, which encodes the MRS6/MSI4 gene product [26,100]. Recent findings have also identified a novel GGTase-III enzyme, a previously uncharacterized human prenyltransferase complex consisting of an orphan prenyltransferase α subunit, PTAR1, and the catalytic β subunit of GGTase-II, Rab-GGTB (Figure 4) [98,101]. This enzyme specifically recognizes the C-terminal CaaX prenylation motif of FBXL2 and mediates its geranylgeranylation. Such modification is crucial for the localization of FBXL2 to cell membranes, where it functions as a ubiquitin ligase to mediate the polyubiquitylation of membrane-anchored proteins.

GGTase-II is responsible for the geranylgeranylation of Rab GTPases (a family of Ras-related small GTPases) in both yeast and animals, which are involved in vesicle transport and organelle biogenesis [26]. Rab-GGT geranylgeranylation modification defects are also related to a variety of human diseases [102]. For instance, patients with choroideremia exhibit loss-of-function mutations in REP1, while the murine model of Hermansky–Pudlak syndrome known as gunmetal carries a splice-site mutation in the α-subunit of GGTase-II [103]. In plants, RABs and SNAREs regulate membrane trafficking, with RABs mediating vesicle/organelles tethering and membrane fusion, generally driven by SNARE complexes (Qa/Qb/Qc- and R-SNAREs) [104]. Notably, ARA6 homologs are found in land plants—including angiosperms, a lycophyte, and a bryophyte—but not in green algae; this has been confirmed by phylogenetic analyses indicating monophyly [93]. ARA7/RHA1 functions in endocytosis and vacuolar transport, partially mirroring the roles of animal RAB5. Although ARA6 retains some structural similarities to conventional RAB GTPases, it lacks their C-terminal hypervariable region as well as the cysteine motif necessary for membrane binding and localization [93]. This observation suggests that ARA6 may not undergo modification via C-terminal prenylation; instead, it likely anchors to membranes through N-terminal fatty acylation. In Arabidopsis, ARA6 is involved in endosomal trafficking and regulates a salt stress-responsive SNARE complex that is distinct from those mediated by conventional RAB5 assemblies, highlighting specific adaptations within plant trafficking mechanisms [93].

Prenylation modification is closely related to diseases such as cancer (such as RAS-driven tumors), progeria (such as HGPS), viral infections (such as HDV), etc. Inhibitors targeting the prenylation pathway (such as FTIs, GGTIs) have potential in disease treatment, but challenges such as cross-modification resistance exist [21]. The complete loss of farnesyltransferase is fatal in yeast or animals [105,106]. In animals, interfering with the farnesylation of Ras has been shown to inhibit tumor growth [107]. Consequently, the farnesylation modification pathway has attracted significant attention, particularly in the context of cancer treatment in humans. The farnesylation of the Ras protein is essential for its localization to the cell membrane, which in turn promotes tumorigenesis. Farnesyltransferase inhibitors (FTIs) can inhibit the farnesylation modification of Ras protein, preventing it from binding to the cell membrane, thereby effectively inhibiting tumor growth and metastasis [108]. Furthermore, several proteins involved in cell cycle regulation, as well as a subset of nuclear lamins, have been identified as farnesylated and are associated with cancer [109] and premature aging [110]. Farnesylation plays an important role in tumor proliferation and metastasis [108], premature aging [111], and neurological diseases [112]. However, small GTP-binding proteins in plants, such as Rab, Rho, Arf, and Ran GTPases, do not include Ras GTPases [69]. Nevertheless, researchers have also identified substrates for farnesylation modifications related to the cell cycle and cell proliferation in plants, which is consistent with previous conclusions that certain biological functions of farnesylation modifications are conserved across both animals and plants. In Arabidopsis, several cell cycle regulators characterized by the CAAX terminal sequence are hypothesized to be farnesylated proteins, including NAP1-1, NAP1-2, NAP1-3, adaptin, and cyclophilin (At2g19480: CKQQ, At4g26110: CKQQ, At5g56950: CKQQ, At2g46520: CGIA, At2g38730: CGEM) [7]. Notably, NAP1-2 (AtNAP1;1) plays a critical role in a regulatory mechanism that connects cell proliferation with cell growth and expansion during the development of Arabidopsis leaves, a process that depends on its farnesylation status [46]. During the cell proliferation phase of leaf development, farnesylated NAP1-2 is localized to the nucleus to facilitate cell division. As later leaf development progresses, non-farnesylated NAP1-2 accumulates in the cytoplasm, where it facilitates cell expansion.

## 5. Challenges and Future Perspectives

Recent advances in protein prenylation research have uncovered its significant roles in plant development, hormonal signaling pathways, and stress adaptation, yet numerous substrates and regulatory mechanisms remain uncharacterized. Although over 119 potential prenylation substrate proteins have been identified in Arabidopsis [7], only a limited number have been experimentally validated. Additionally, the influence of environmental factors such as light, temperature, and nutrient availability on prenylation dynamics is not yet fully understood. Omics data can be utilized to identify protein prenylation processes, including genomics, proteomics, lipidomics, and bioinformatics approaches. For example, genomic data can be screened for genes containing CaaX motifs or CC, CXC motifs to identify potential prenylated protein genes [21,113]; proteomics enables the enrichment and identification of prenylated proteins from complex samples to validate their modification status, employing techniques such as biological labeling, antibody enrichment, and mass spectrometry (MS) analysis [113]; isotopic tracing using ^13^C-labeled MVA or MEP pathway precursors allows tracking of isoprenoid flux [44]; additionally, databases can be leveraged to predict prenylated proteins, while KEGG pathway analysis helps elucidate functional networks associated with prenylated proteins. Future investigations integrating omics data and imaging techniques are anticipated to facilitate substrate identification and enhance our comprehension of this post-translational modification.

Manipulation of protein prenylation presents a promising avenue for crop improvement. Current studies have demonstrated the potential of gene knockout strategies, such as *era1*, to improve drought resilience in canola (*Brassica napus* L.) plants [114]. In rice, overexpression of *OsRab7*, a geranylgeranylated GTPase (LOC_Os05g44050.1, containing a SGCC C-terminal sequence), enhances salt tolerance through regulation of vacuolar trafficking, illustrating the applicability of prenylation targets in agricultural improvement [115]. Furthermore, targeted disruption of *RAM1* in the rice blast fungus *Magnaporthe oryzae* reduces hyphal growth and sporulation while increasing sensitivity to multiple stresses [116]. Notably, loss of *RAM1* also attenuates virulence in plant hosts, suggesting that inhibiting farnesyltransferase activity may serve as an effective strategy for enhancing pathogen resistance [116]. By modulating the expression or activity of prenylation enzymes and their substrates, it may be feasible to enhance plant growth, development, and stress tolerance, ultimately contributing to increased crop yields and improved agricultural sustainability.

## 6. Conclusions

Protein prenylation is a conserved post-translational modification that profoundly influences plant growth, integrating developmental programs and stress adaptability. In plants, this process is mediated by three primary prenyltransferases—FTase, GGTase-I, and GGTase-II—each recognizing distinct substrate motifs and facilitating protein membrane association through farnesyl or geranylgeranyl lipid anchors. Prenylation modulates critical cellular processes, including hormone signaling (e.g., ABA and auxin), vesicle trafficking, and cytoskeletal dynamics, while playing crucial roles in both abiotic (drought, salt, heat, heavy metals) and biotic (fungal, bacterial, viral) stress responses. Evolutionarily, plant prenylation mechanisms share core enzymatic machinery with other eukaryotes but exhibit species-specific adaptations. Biotechnologically, targeting prenylation enzymes (e.g., FTase inhibition) or their substrates (e.g., *OsRab7* overexpression) holds promise for developing stress-resilient crops. However, challenges persist in fully deciphering substrate specificity, regulatory networks, and environmental influences on prenylation dynamics. Future research integrating omics and imaging techniques will likely unravel remaining mysteries, solidifying protein prenylation as a key target for enhancing plant fitness and agricultural sustainability.

## Data Availability

No new data were created or analyzed in this study.
